# Detection of *Bartonella vinsonii* subsp. *berkhoffii* in an HIV patient using metagenomic Next-Generation Sequencing

**DOI:** 10.1080/22221751.2022.2094287

**Published:** 2022-07-17

**Authors:** Yuru Shi, Jing Yang, Yingjie Qi, Junlan Xu, Yingqi Shi, Tiantian Shi, Chao Liu, Xiaoling Ma

**Affiliations:** a Department of Clinical Laboratory, Infection Hospital Area of the First Affiliated Hospital of University of Science and Technology of China, Hefei, People’s Republic of China; b Medical Department, Hangzhou Matridx Biotechnology Co., Ltd, Hangzhou, People’s Republic of China

**Keywords:** *Bartonella vinsonii*, AIDS, metagenomic Next-Generation Sequencing, bacillary angiomatosis

## Abstract

Bartonella species are fastidious, aerobic bacteria that are transmitted by blood-sucking arthropods. *Bartonella* spp. are responsible for cat scratch disease, Carrion’s disease, bacillary angiomatosis and trench fever. On the other hand, *Bartonella vinsonii* is rarely reported in the literature and there exist a few reports of systemic infection caused by *Bartonella vinsonii* in patients with acquired immunodeficiency syndrome. A 31-year-old male (diagnosed with AIDS six years ago) had persistent fever and ulceration in the right knee. The elevated levels of inflammatory markers suggested an infectious aetiology. Despite the negative findings of blood culture, metagenomic Next-Generation Sequencing of plasma detected *Bartonella vinsonii*. The polymerase chain reaction of whole blood and Sanger sequencing confirmed the mNGS findings. Immunohistochemical staining had later suggested bacillary angiomatosis, which was consistent with Bartonella infection. Following antibiotic treatment, the ulcers subsided significantly, but a high fever persisted. The patient died due to sudden respiratory failure.

*Bartonella* spp. are fastidious gram-negative bacilli that require an enriched culture medium (which contains haeme) and a high CO_2_ level for growth [1,2]. They can cause lymphadenitis, endocarditis, encephalitis, visceral abscessesfever in immunocompetent individuals and bacillary angiomatosis in immunocompromised patients [3]. *Bartonella henselae* is the most reported member of the Bartonella species, which are transmitted to humans through bites and scratches by cats [4,5]. On the other hand, infectious diseases due to *Bartonella vinsonii* are rare and are typically carried by canines [6]. Here we present a case of *Bartonella vinsonii* subsp. *berkhoffii* infection that led to bacillary angiomatosis in a patient with AIDS, which was detected by mNGS and confirmed by PCR and pathological staining.

The patient (male, Han Chinese, 31 years old) was a farmer and owned a pet dog. Six years ago, the patient was diagnosed with acquired immunodeficiency syndrome (AIDS) and had since been taking HIV medicines irregularly. The CD4+ T cell count of the patient dropped from 500 to 95/mL (from June to December 2021). One month before admission, the patient noticed a red spot on the right knee, which progressed into a lump (Figure 1(A)). The patient had a fever, weight loss (decreased by 5 kg from September 2021 to January 2022) and right knee ulcers for 21 days, accompanied by eating difficulties and a feeling of choking. Upon admission, granuloma and swelling with a height of 2–3 cm were seen on the right knee. The texture was brittle that bled easily. A dark brown rash was seen across the torso (Figure 1(B, C)). The patient tested positive for *Treponema pallidum* and was emaciated. The routine laboratory test results can be found in Table 1, which showed haemoglobin (56 g/L), red blood cell (1.92 × 10^12^/L), platelet (5.0 × 10^9^/L) and CD4+ T cell count (5 cells/µl) were significantly reduced. In addition, inflammatory markers, including CRP (94.25 mg/L) and PCT (4.98 ng/mL), were elevated. On the other hand, T-SPOT.TB, serum (1,3)-β-D glucan test, galactomannan test and blood culture were all negative. Due to severe anaemia, the patient was given intravenous infusions of red blood cells. Moreover, lamivudine, dotiravir and tenofovir fumarate were administered as anti-HIV treatment. Meropenem was empirically used as a broad-spectrum antibiotic. The plasma samples were sent for mNGS testing (detailed methods can be found in one of our previous publications [7]) on January 12th, which reported *Bartonella vinsonii* (2352 reads), EBV (1 read) and CMV (1 read) (Figure 1(D)). Pleural effusion was tested on January 27th, which again reported *Bartonella vinsonii* (3 reads) and EBV (1 read). Both mNGS tests returned results in less than 24 h (sequencing files were deposited into the NCBI SRA database and can be retrieved at https://www.ncbi.nlm.nih.gov/sra with accession number PRJNA821973). The subspecies of *Bartonella vinsonii* was *berkhoffii* as we detected 69 and 2 uniquely mapped reads from plasma and pleural effusion, respectively. To further confirm the microbiological findings, we designed primers that specifically targeted *Bartonella vinsonii* and performed PCR (forward 5’-GCAGGCATAACGAAGCACAT-3’ and reverse 5’-CCCAGCTGTCCATCATTCCA-3’) using total DNA extracted from 200 μL of whole blood (95°C 3 min, followed by 40 cycles of 95°C 5s, 60°C 15s). Peripheral blood collected from a healthy individual was used as a negative control. The PCR amplicons were then sent for Sanger sequencing. As expected, the patient sample, rather than the negative control, showed specific amplification of *Bartonella vinsonii* (Figure 1(E, F)).

**Figure 1.**
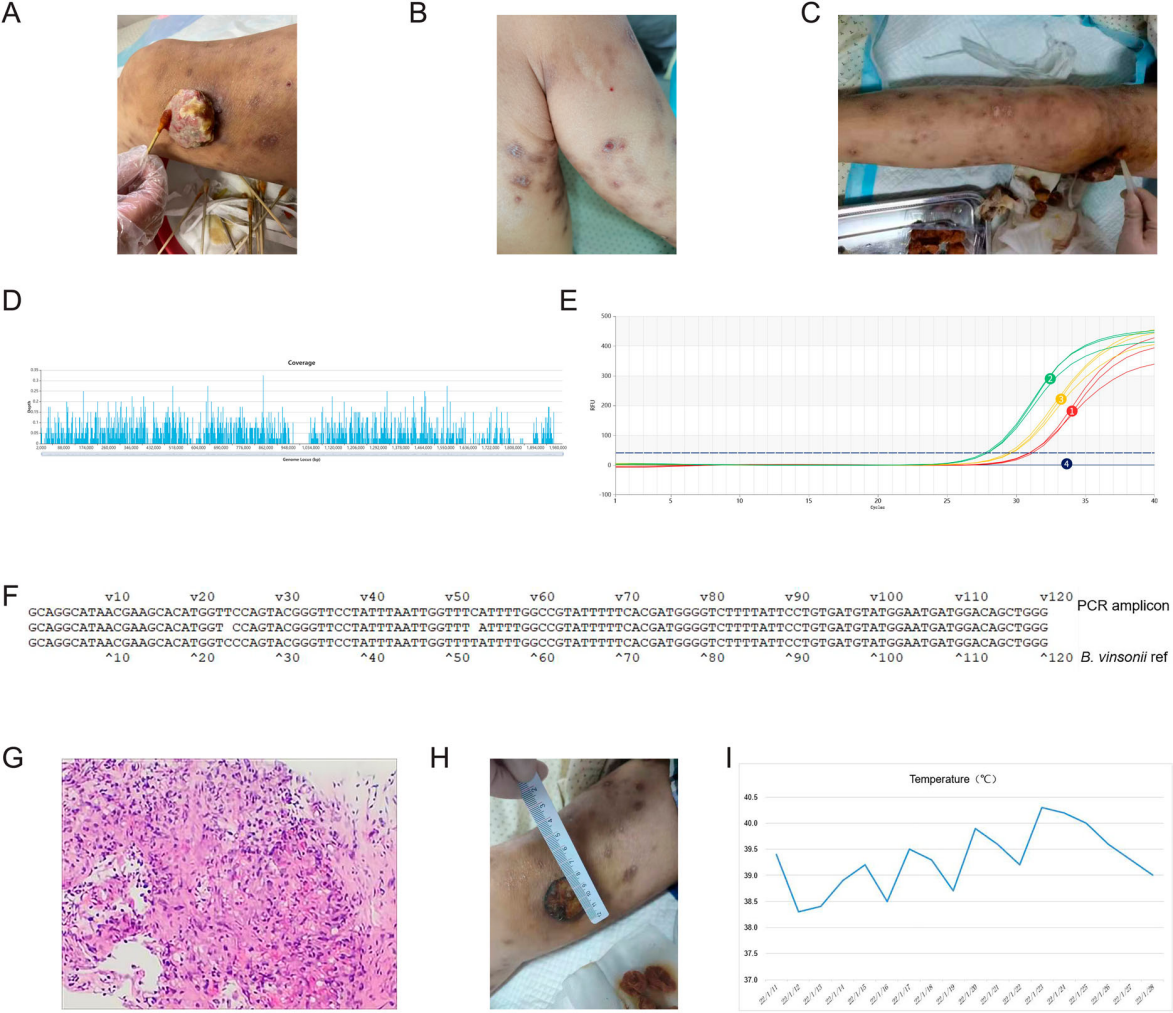
The diagnosis and treatment of *Bartonella vinsonii* infection. The lesion around the right knee was shown in (A), and red spots were seen on the right (B) and left leg (C) of the patient. mNGS results were shown in (D) and fluorescent PCR results were displayed in (E), in which blood samples collected from days 11, 13 and 14 after admission were shown as amplification curves 1 (red), 2 (green) and 3 (orange), respectively. Negative control was shown as curve 4 (dark blue). Sanger sequencing alignment was shown in (F). Haematoxylin and eosin (H&E) staining of biopsy tissue was shown in (G). Improvement of the right knee lesion was shown in (H) and the temperature chart of the patient during hospitalization was shown in (I).

**Table 1.** Laboratory results of the patient after admission.

Laboratory Test	Results	Reference Value
WBC	5.59 × 10^9^/L	3.5–9.5 × 10^9^/L
Neutrophil count	3.96 × 10^9^/L (71%)	40–75%
Lymphocyte count	0.84 × 10^9^/L (15%)	20–50%
Monocyte count	0.77 × 10^9^/L (13.7%)	3–10%
Haemoglobin	56 g/L	130–175 g/L
RBC	1.92 × 10^12^/L	4.3–5.8^12^/L
Platelet count	5.0 × 10^9^/L	125–350 × 10^9^/L
C-reactive protein	94.25 mg/L	0–8 mg/L
Procalcitonin	4.98 ng/mL	0–0.5 ng/mL
HIV serology	Positive	Negative
*Treponema pallidum* antibody	Positive	Negative
CD4 absolute count	5 cells/µL	410–1590 cells/µL
T-SPOT.TB	Negative	Negative
G test	Negative	Negative
GM test	Negative	Negative
Blood culture	Negative	Negative

To confirm the microbiological findings, a biopsy of knee ulcers was collected under local anaesthesia. The outer upper edge of the mass was chosen as the puncture point. The samples were sent for pathology tests. One week later, immunohistochemical staining had shown cells positive for SMA, Desmin, ERG, CD31, CD34 and Ki-67 value-added index was 15% (this value describes how many cells are dividing, 20% or higher is considered high). Meanwhile, HHV8, CK, and S100 were negative. These results suggested that the lesion was angiogenic and not likely to be a malignant tumour. Notably, Warthin-Starry staining was positive, which pointed to bacillary angiomatosis (Figure 1(G)). Taken together, these observations were consistent with *Bartonella vinsonii* infection. Therefore, azithromycin, doxycycline and rifampin were used*.* The mass shrunk significantly, and the rash subsided (Figure 1(H)). Haemoglobin and platelet count were also rising, but high fever persisted despite antibiotic treatment (Figure 1(I)). On day 16, the patient had chest tightness and shortness of breath. On day 17, the patient had chills, fever, elevated heart rate (120 bpm), shallow and fast breathing, mild cyanosis on the lips and 78– 80% oxygen saturation. On day 18, the patient had respiratory failure and relapsed into a coma before his death.

Bartonella can cause a spectrum of clinical symptoms, including fever, hallucinations, weight loss, muscle fatigue, partial paralysis, endocarditis [8], neuropsychiatric syndrome and other neurological manifestations [6], making differential diagnosis extremely difficult. The microbiological culture of Bartonella species is also challenging. Therefore, detection of *Bartonella* spp. is dependent on serology, tissue staining (*i.e.* Warthin-starry) and molecular tests. However, these tests are targeted and thus require *a priori* assumptions. On the other hand, mNGS can unbiasedly sequence all potential pathogens directly from clinical samples [9].

In this case, the patient was immunocompromised due to AIDS. An Infectious aetiology was suspected but with no backing from traditional microbiological tests. The tumour was also considered and therefore biopsies were taken for pathological examination. While waiting for the results, plasma mNGS reported *Bartonella vinsonii* subsp. *berkhoffii*. This finding was plausible as the patient owned a pet dog, which is known to carry and transmit *Bartonella vinsonii* [10]. The association between Bartonella infection and bacillary angiomatosis was first reported in 1990 [11], while the infection of *Bartonella* spp. in AIDS patients was first reported in 1998, which described bacillary angiomatosis that was characterized by neovascular proliferation in the skin due to infections by *Bartonella quintana* and *Bartonella henselae*. Moreover, proliferative vascular diseases caused by *Bartonella vinsonii* subsp. *berkhoffii* have been reported [12]. In our case, the patient had bacillary angiomatosis. Additionally, the patient had anaemia, thrombocytopenia, rapid decline of CD4 + T cells and high fever. He experienced eating difficulties and a feeling of choking, which might be suggestive of dysfunctions of the autonomic nervous system that was caused by Bartonella infection. According to previous studies, a combination of doxycycline and rifampin is recommended for the treatment of Bartonella infection, especially in patients with severe diseases [13]. Owing to mNGS, targeted antibiotics were applied without much delay, which led to increases in red blood cell and platelet count and a decline in inflammatory marker levels. On the other hand, Murillo *et al* reported a 44-year-old HIV patient presented with a mass on the lateral side of the neck [14]. Due to negative microbiological findings, tuberculous was suspected but the anti-tuberculosis treatment had caused deterioration of symptoms and death of the patient. Only during the autopsy, the patient was found to be infected with *Bartonella quintana.* Unfortunately, in our case, an autopsy was not performed, and the exact cause of respiratory failure was not established. Although the patient also died in our case, we still wanted to point out the importance of a fast and accurate microbiological result in diagnosing and treating *Bartonella* infections.
